# The well-being of mothers caregiving for children with cerebral palsy: a Tunisian cross-sectional study

**DOI:** 10.11604/pamj.2025.51.33.45979

**Published:** 2025-06-07

**Authors:** Ikram Haddada, Soumaya El Arem, Aymen Hadj Salah, Manel Ben Fredj, Aymen Fekih, Tammem Sayhi, Mouhamed Hedi Zaafrane, Mouna Sghir, Wassia Kessomtini

**Affiliations:** 1 Physical Medicine and Rehabilitation Department, Tahar Sfar University Hospital, Mahdia, Tunisia; 2 Department of Epidemiology and Preventive Medicine, Fattouma Bourguiba University Hospital of Monastir, Monastir, Tunisia; 3 Department of Trauma and Orthopaedics Surgery, Fattouma Bourguiba University Hospital of Monastir, Monastir, Tunisia; 4 Department of Family Medicine, University of Monastir, Monastir, Tunisia

**Keywords:** Cerebral palsy, caregivers, sleep disorders, caregiver burden, fatigue, anxiety

## Abstract

**Introduction:**

cerebral palsy (CP) is the most common physical disability in childhood. Caring for a child with functional limitations and long-term dependence poses significant challenges for many families. Despite this, the experiences and needs of mothers of children with CP remain understudied. This study aims to assess fatigue, caregiving burden, psychological distress, and sleep disorders (SDs) among a group of Tunisian mothers of children with CP.

**Methods:**

this cross-sectional study explored the well-being of Tunisian mothers caring for children with CP. Participants were evaluated using the Checklist Individual Strength (CIS), Zarit Caregiver Burden Scale (ZCBS), Hospital Anxiety and Depression Scale (HADS), and Pittsburgh Sleep Quality Index (PSQI).

**Results:**

a total of 71 participants were recruited. Of all participants, 39.4% reported moderate to severe burden, and 53.5% reported severe fatigue. The prevalence of clinically significant depressive or anxious symptoms was found to be 63.4% and 53.5%, respectively. The prevalence of SDs was 74.6%. The most affected components of the PSQI were sleep latency, sleep quality, and sleep dysfunction. Sleep quality was significantly related to epilepsy in children (p=0.012), mothers' age (p=0.027), mothers' depression (p=0.008), or anxiety (p=0.001). No significant association was found between mothers' burden or fatigue and low sleep quality.

**Conclusion:**

our findings highlight the prevalence of fatigue, burden, serious psychological distress, and SDs among mothers of children with CP. Consequently, medical personnel and social workers should be more attentive to the needs of these mothers who often silently endure the emotional and physical strains of caregiving.

## Introduction

Cerebral palsy (CP) is a term used to describe a group of lifelong disorders of mobility and posture that limit activities and are caused by nonprogressive disturbances in the developing foetus or infant brain [1]. The overall prevalence of CP has remained stable at around 2.1 per 1000 live births [2]; however, it is lower in high-income countries where the prevalence is declining with ranges from 1.4 to 1.7 per 1000 live births [3]. In Tunisia, there is a significant lack of information regarding the prevalence of CP [4].

The motor disorders of CP are often accompanied by sensory, perceptual, cognitive, and communication disturbances, as well as epilepsy and subsequent musculoskeletal problems [1]. As a result, children with CP often require external assistance to perform daily activities, which is frequently provided by parents who become primary caregivers [5]. In developing countries like Tunisia, the most chronically ill children, such as children with CP, are primarily cared for by their mothers.

Typically, caregiving for a child with CP involves a wide range of activities, such as heavy lifting, turning, bathing, toileting assistance, bedtime routines, dressing, and transfers [5]. Thus, providing care for a child with functional limitations and long-term dependence is an entirely burdensome activity and may be physically and emotionally exhausting for mothers [6]. In some cases, providing such care can lead to stress, anxiety, depression, fatigue, and impaired sleep quality [5].

Sufficient sleep is essential for optimal physical and mental health [7]. Consequently, poor sleep among caregivers may increase their risk of cardiovascular and metabolic diseases, as well as psychological problems and decreased energy levels [8]. Despite its significance, the physical and psychological well-being of mothers of children with CP in North African and Arab populations remains understudied.

While several global studies have examined common caregiver challenges such as care burden, elevated rates of anxiety or depression, sleep disorders (SDs), and fatigue in mothers of children with CP separately, relatively few have comprehensively investigated all these factors within the same patient group [5]. To address this gap, the objectives of this study are twofold: (1) to evaluate fatigue, care burden, psychological distress (anxiety and depression), and SDs among a group of Tunisian mothers of children with cerebral palsy; (2) to determine the associated factors of poor sleep quality.

## Methods

**Study design:** this cross-sectional study employed an exhaustive data collection approach, enrolling all mothers of children with CP who attended routine consultations at our Tunisian physical medicine department between September 2023 and June 2024.

**Study setting:** this study took place at a specialized Physical Medicine Department in Tunisia, a North African country with extremely limited national resources in this medical field [9]. The center where the research was conducted is the only specialized physical medicine and rehabilitation facility serving its region. As such, it's the sole referral center for pediatric rehabilitation in the area, providing comprehensive care for children with CP, from diagnosis to therapeutic interventions and long-term follow-up.

**Participants:** the study included mothers who were the primary caregivers for children aged four or younger with a diagnosis of CP, confirmed by a pediatrician, pediatric neurologist, or physiatrist. The following criteria were used for exclusion: (a) Mothers who were unable to complete the questionnaire; (b) mothers caring for another child under the age of two; (c) mothers with another child at home who has special healthcare needs; (d) mothers providing care for an elderly, chronically ill, or disabled relative; (e) mothers with a history of chronic illness, such as diabetes, pulmonary disease, kidney disease, musculoskeletal diseases, or any psychiatric disorder that began before the child's diagnosis of CP.

**Ethical considerations:** ethical approval for this study was secured from the relevant committee. During their child's routine clinical visits, eligible mothers were verbally informed about the study's purpose, procedures (including questionnaires and time requirements), and its voluntary and anonymous nature by a treating physician, who also provided a written information sheet. Following these explanations of their rights, including the freedom to refuse participation without affecting their child's care and the ability to withdraw at any time, written informed consent was obtained from all participants, adhering to standard ethical approval procedures.

**Instrument:** the demographic characteristics of the children (age and gender) were recorded. The severity of a child's motor disability was assessed using the Gross Motor Function Classification System (GMFCS) [10]. The GMFCS is a standardized tool designed to describe gross motor function in children with CP. It focuses on self-initiated movements, such as sitting and walking. The system is divided into five levels, with level I representing the least limitation and level V the greatest limitation. Motor distribution of CP was classified as monoplegia, diplegia, hemiplegia, and quadriplegia. The motor type of CP was classified as spasticity, dyskinesia, ataxia, and hypertonia. The presence of associated conditions, including visual impairment, hearing impairment, language delay, epilepsy, pain, lower urinary tract symptoms (LUTS), or psychological problems, was noted.

Co-sleeping, defined as the child sleeping in either the same room or the same bed as the caregiver, was also documented. Sociodemographic data of the participants (age, educational level, marital status, number of children, occupation, and type of residence) were included in the questionnaire. According to the 2015 criteria of the National Institute of Statistics (Tunisia) [11], participants were classified socioeconomically.

**Measure of care burden:** the Zarit Caregiver Burden Scale (ZCBS) was used to evaluate the care burden experienced by all participants [12]. The ZCBS is an instrument designed to measure the stress associated with providing family care. It consists of 22 questions covering various aspects of health, social life, personal life, financial situation, emotional well-being, and interpersonal relations. Each item on the ZCBS is rated on a 5-point Likert scale, ranging from 0 = “never” to 4 = “nearly always”. The scores for all items are summed to calculate a total score, which ranges from 0 to 88. Higher scores indicate a greater level of caregiver burden. According to the ZCBS scoring guidelines, a total score of 0-20 indicates “no care burden”, 21-40 indicates “light care burden”, 41-60 indicates “moderate care burden”, and 61-88 indicates “heavy care burden” [13].

**Measure of fatigue:** the Multidimensional Checklist Individual Strength (CIS) was used to measure chronic fatigue. The CIS consists of 20 statements that are rated on a 7-point Likert scale [14]. The tool is divided into four dimensions: (a) subjective experience of fatigue (8 items); (b) reduced motivation (4 items); (c) reduced activity (3 items); and (d) reduced concentration (5 items). A total CIS score is calculated by summing the scores from these four dimensions. The possible range for the total score is 20 to 140, with higher scores indicating a higher degree of fatigue, more concentration problems, lower motivation, and less activity. According to the CIS scoring guidelines, an overall score of ≤ 26 indicates “no fatigue in caregivers”, 27-34 indicates “mild fatigue”, and ≥ 35 indicates “severe fatigue” [15].

**Psychological well-being:** the Hospital Anxiety and Depression Scale (HADS) [16] is a self-report questionnaire designed to screen for emotional distress in non-psychiatric patients. It effectively identifies both depression and anxiety. The HADS consists of two subscales (anxiety and depression), each with seven items. Each item is rated on a scale of 0-3, and the scores for all items are summed to calculate a total score ranging from 0 to 21. Higher scores indicate greater levels of anxiety and depression. A score of 11 or higher on either the depression or anxiety subscale is considered to indicate clinically significant symptoms of that disorder (i.e., probable cases of depression or anxiety). Scores between 8 and 10 are considered borderline cases, while scores below 8 are considered normal [17].

**Measure of sleep quality:** the Pittsburgh Sleep Quality Index (PSQI) [18] was used to assess caregiver sleep quality during the four weeks prior to the survey. The PSQI is a self-report measure consisting of 19 items that evaluate sleep quality in seven components: subjective sleep quality (1 item), sleep latency (2 items), sleep duration (1 item), habitual sleep efficiency (3 items), sleep disturbances (9 items), use of sleep medication (1 item), and daytime functioning (2 items). Each component is scored on a scale of 0 to 3, with 0 indicating no sleep problem and 3 indicating severe sleep difficulties. The scores for all components are summed to produce a total PSQI score ranging from 0 to 21. Based on this total score, sleep quality is classified as good (0-5 points) or poor (6-21 points) [5].

**Statistical analyses:** statistical analysis was conducted using SPSS, version 26. Descriptive statistics were used to characterize the initial assessments of fatigue, care burden, depression, anxiety, and sleep disturbances among mothers of children with CP. This involved presenting means with standard deviations for quantitative data and numbers with proportions for qualitative data. The Kolmogorov-Smirnov test was employed to assess the normality of data distribution.

To achieve the study's second objective of identifying factors associated with poor sleep quality, we defined poor sleep quality in mothers of children with CP as the dependent variable, operationalized by a PSQI score ≥ 5. We then investigated two distinct categories of explanatory variables: child-related factors (including gender, age, CP type, CP motor distribution, GMFCS level, co-sleeping status, daily pain, and associated conditions like epilepsy) and maternal factors (comprising age, level of fatigue, care burden, and indicators of psychological well-being). Chi-square tests were used to compare percentages, and Student's t-tests were utilized for comparing means between groups. Finally, a binary logistic regression analysis was performed to identify the primary independent variables linked to poor sleep quality among mothers of children with CP (our dependent variable). Statistical significance for all analyses was set at p < 0.05.

## Results

The number of eligible individuals who participated during the data collection period was 71.

**Demographics characteristics of caregivers:** the mean age of caregivers was 38.77 years (SD: 7.27 years). Thirty-nine (54.9%) mothers were aged 35 to 44 years. The majority of participants (93%) were married, with over two-thirds (69%) being housewives. More than half (53.5%) of caregivers resided in urban environments. Socioeconomic analysis indicated that 45.1% of caregivers belonged to the lower socioeconomic class. A substantial number (57.7%) had completed at least a secondary level of education. Furthermore, 46.5% of mothers had at least two other children in addition to their child with CP.

**Demographics and clinical characteristics of children with CP:** the mean age of children was 7.7 years (SD: 2.75 years). Eighteen (25.4%) children were in the age range of 4 to 6 years. There were 39 (54.9%) male children, resulting in a male-to-female ratio of 1.2. Thirty-eight (53.5%) of the children had severe deficits (GMFCS IV: 18.3% and GMFCS V: 35.2%). Demographic and clinical data of children with CP are shown in Table 1.

**Table 1:** demographic and clinical characteristics of the children with cerebral palsy

Characteristics	n=71 (%)
Sex males/females	39/32 (54.9/45.1)
**Age group (years)**	
< 6	18 (25.4)
≥ 6	53 (74.6)
**Co-sleeping**	
Room sharing at night	16 (22.5)
Bed sharing	30 (42.2)
**Type of cerebral palsy**	
Spasticity	67 (94.4)
Dyskinesia	1 (1.4)
Ataxia	3 (4.2)
**CP motor distribution**	
Diplegia	13 (18.3)
Hemiplegia	20 (28.2)
Quadriplegia	34 (47.9)
Monoplegia	4 (5.6)
**Motor impairment (GMFCS)**	
I	7 (9.9)
II	11 (15.5)
III	15 (21.1)
IV	13 (18.3)
V	25 (35.2)
**Associated conditions**	
Epilepsy	34 (47.9)
Hearing impairment	8 (11.3)
Visual impairment	21 (29.6)
Language delay	55 (77.5)
Psychological problems	13 (18.3)
LUTS	27 (38)
**Report experiencing pain daily**	
Yes	34 (47.9)
No	37 (52.1)

CP: cerebral palsy; GMFCS: gross motor function classification system; LUTS: lower urinary tract symptoms

**Prevalence of care burden, fatigue, and psychological distress among caregivers:** as illustrated in Table 2, twenty-eight (39.4%) participants reported moderate to severe burden (ZCBS ≥ 41), and 38 (53.5%) of caregivers were severely fatigued (CIS ≥ 35). Using the established cut-offs on the HADS, the prevalence of clinically significant depressive symptoms (HADS depression scale ≥ 11) was found to be 63.4%. Regarding anxiety, clinically significant symptoms (HADS anxiety scale ≥ 11) were found in 53.5% of participants.

**Table 2:** means and distribution of depression, anxiety, fatigue, and caregiver burden scores among mothers of children with cerebral palsy

	n=71 (%)
ZCBS (mean ± SD)	36.43 ± 13.9
**Mother’s Burden**	
No care burden	11 (15.5)
Light care burden	32 (45.1)
Moderate care burden	27 (38)
Heavy care burden	1 (1.4)
CIS (mean ± SD)	33.8 ± 8.3
**Fatigue in mothers**	
No fatigue	15 (21.1)
Mild fatigue	18 (25.4)
Severe fatigue	38 (53.5)
HADS depression scale (mean ± SD)	11.24 ± 3.46
**Depression symptoms**	
Little or none (HADS <8)	11 (15.5)
Probable/Possible (HADS 8-10)	15 (21.1)
Significant symptoms (HADS ≥ 11)	45 (63.4)
HADS anxiety scale (mean ± SD)	10.72 ± 4.66
**Anxious symptoms**	
Little or none (HADS <8)	17 (24)
Probable/Possible (HADS 8-10)	16 (22.5)
Significant symptoms (HADS ≥ 11)	38 (53.5)

CIS: checklist individual strength; HADS: hospital anxiety and depression scale; SD: standard deviation; ZCBS: Zarit caregiver burden scale

**Sleep disturbances among caregivers and associated factors:** the PSQI global score of the sample ranged from 0 to 17. The mean PSQI score of the study mothers was 7.6 ± 4.2, with fifty-three (74.6%) having a PSQI score of ≥ 5, indicating SDs and poor quality of sleep. Twenty-seven (38%) participants were classified as having a short sleep duration (< 6 hours/day). Of the seven components, the most affected were sleep latency, sleep quality, and sleep dysfunction.

Regarding our second objective, which sought to identify factors linked to poor sleep quality in mothers, participants were categorized as good sleepers (PSQI < 5) or poor sleepers (PSQI ≥ 5) based on their global PSQI scores. As indicated in Table 3, maternal sleep quality showed significant associations with several factors: the presence of epilepsy in their children (p=0.012), the mothers’ age (p=0.027), and their levels of depression (p=0.008) and anxiety (p=0.001). Notably, the severity of a child’s motor disability (GMFCS level) was not found to be a factor associated with the mothers’ sleep quality, and no significant association was observed between mothers’ burden or fatigue and low sleep quality.

**Table 3:** factors influencing sleep quality in mothers of children with cerebral palsy

Variable	Good sleeper (n=18) (25.4%)	Poor sleeper (n=53) (74.6%)	P-value
**Demographic and clinical characteristics of the children with CP**			
Gender n (%)			
Males	12 (30.8)	27 (69.2)	
Females	6 (18.8)	26 (81.3)	0.24
**Age category n (%)**			
< 6 years	6 (33.3)	12 (66.7)	0.36
≥ 6 years	12 (22.6)	41 (77.4)	
**CP type n (%)**			
Spastic	18 (26.9)	49 (73.1)	0.23
Non-spastic	0 (0)	4 (100)	
**CP motor distribution n (%)**			
Hemiplegia/ monoplegia	8 (33.3)	16 (66.7)	0.27
Quadriplegia/ diplegia	10 (21.3)	37 (78.7)	
**GMFCS level n (%)**			
I, II, III	8 (24.2)	25 (75.8)	0.84
IV, V	10 (26.3)	28 (73.7)	
Co-sleeping n (%)	10 (21.7)	36 (78.3)	0.34
Report experiencing pain daily n (%)	11(29.7)	26 (70.3)	0.37
Epilepsy n (%)	4 (11.8)	30 (88.2)	0.012
Hearing impairment n (%)	1 (12.5)	7 (87.5)	0.37
Visual impairment n (%)	7 (33.3)	14 (66.7)	0.31
Language delay n (%)	15 (27.3)	40 (72.7)	0.49
Psychological problems n (%)	5 (38.5)	8 (61.5)	0.22
LUTS n (%)	8 (29.6)	19 (70.4)	0.51
**Characteristics of mothers**			
**Age category n (%)**			
< 35 years	8 (47.1)	9 (52.9)	0.027
≥ 35 years	10 (18.5)	44 (81.5)	
ZCBS (mean ± SD)	36.83 (±10.65)	36.3 (±15.05)	0.89
CIS (mean ± SD)	34.5 (±9.28)	33.66 (±8.04)	0.71
HADS depression scale (mean ± SD)	9.06 (± 3.95)	11.98 (±3)	0.008
HADS anxiety scale (mean ± SD)	8.56 (± 4.26)	11.45 (±4.6)	0.001

CP: cerebral palsy; CIS: checklist individual strength; HADS: hospital anxiety and depression scale; LUTS: lower urinary tract symptoms; SD: standard deviation; ZCBS: Zarit caregiver burden scale

## Discussion

This study sheds light on the significant challenges faced by mothers caring for children with CP. Our findings indicate a substantial burden on these caregivers: over one-third (39.4%) reported moderate to severe levels of care burden, and more than half (53.5%) experienced severe fatigue. The prevalence of clinically significant depressive or anxious symptoms was found to be 63.4% and 53.5%, respectively. Furthermore, SDs were commonly observed among participants, underscoring a critical aspect of their well-being. To our knowledge, this is the first study to specifically investigate psychological distress, care burden, fatigue, and sleep quality in caregivers of children with CP within an Arab or North African population. This directly addresses a significant geographical and cultural gap in the existing research, providing much-needed insights into the experiences of caregivers in this region. Moreover, by offering a comprehensive assessment of key well-being indicators (including fatigue, anxiety, depression, care burden, and sleep quality), this study provides a holistic understanding of the multifaceted challenges these family caregivers, typically mothers, endure. Notably, it also stands out as one of the few studies to specifically evaluate fatigue in mothers of children with CP, thereby filling another significant gap in the current literature.

The impact of caring for a child with CP on mothers' well-being has been widely investigated [5,19,20], with numerous studies specifically examining their sleep quality [21-24]. These studies consistently report high rates of SDs among mothers of children with CP, with prevalence estimates ranging from 40% to 78% [23]. Our findings align with this existing research, showing that 74.64% of mothers in our study experienced poor sleep quality. Children with physical disabilities, such as CP, are more likely to have sleep problems and require nighttime parental attention [25]. This can contribute to greater sleep disturbance for caregivers [26]. Mothers who experience the highest number of sleep disruptions often have children with the most significant care needs [21].

The principal pediatric factor associated with sleep disturbance in our study was the presence of epilepsy. One of the most direct and significant effects of childhood epilepsy on parental caregivers' physical health is decreased sleep quality. A previous study showed that nearly half of the parents of children with epilepsy have poor sleep quality [27,28]. Parents who care for an epileptic child often experience emotional distress and a disruption in their daily routine. These caregivers need to remain vigilant and provide constant monitoring due to the fear that the child may have a seizure at any time [29].

The present study observed a significant association between the mother's age and low sleep quality. Sleep structure, duration, and quality can be altered in many conditions, particularly with aging. Melatonin deficiency is a major cause of sleep disruption in older populations [30]. In addition to physiological and hormonal factors that may influence sleep changes in elderly caregivers, older mothers are more likely to report depressive symptoms, feel pessimistic about their child's future [31], and experience anxiety about aging and death [32]. Excessive stress has been shown to lead to insomnia and sleep disturbance [33].

Our study aimed to evaluate anxiety and depression symptoms in primary parents of children with CP. The findings indicate that both depression and anxiety are prevalent among these caregivers, with approximately half or more of the participants exhibiting symptoms of these psychological disorders. A review of the related literature and published findings suggests that parents of children with CP tend to experience higher levels of distress and poorer mental health, and that anxiety and depression are the most common mental health disorders in this population [34].

Among the factors that contribute to the development of anxiety and depression in caregivers, the stressful routine they face should be emphasized. The long-term responsibility of caring for a disabled child, combined with the need to overcome adversities and challenges associated with raising the child, often leads to negative emotions such as anxiety, sadness, anger, hopelessness, and distress. These emotions can adversely affect the functioning of the parents and the entire family [35]. However, the intensity of anxiety and depressive symptoms in parents is not solely related to factors associated with the child's disability but is also linked to personal, social, and economic variables resulting from the parents' own needs and health status [36]. Furthermore, these caregivers participate less in social activities, face more challenges at work, and report a higher frequency of family conflicts [37]. Anxiety and depression are often associated with SDs. Anxiety frequently leads to sleep-onset insomnia, while depression is more commonly associated with sleep-maintenance insomnia and early awakening [38]. The present study demonstrated a significant association between psychological distress (anxiety and depression) among caregivers and their sleep quality.

Children with CP primarily experience motor and functional impairments [10]. As a result, mothers provide active support in areas such as feeding, bathing, dressing, ambulation, and transfers. These daily tasks are ongoing and can be demanding [5]. Additionally, some children with CP may require more attention due to additional health challenges like epilepsy, behavioral disorders, non-oral feeding, and SDs [37]. When the care demands for a chronically ill child exceed a certain threshold, it can lead to negative consequences, including stress and caregiver burden [6]. In the present study, we found that 39.4% of participants reported moderate to severe burden. According to the available literature, the prevalence of burden in this sample is higher than the results from another study conducted in a sample of Mexican caregivers, where 30% of participants reported an intense burden [39]. Our findings may be explained by Tunisian beliefs and socio-cultural factors. Mothers of children with CP are often left alone to care for their disabled child due to a lack of spousal support in caregiving. This means that mothers have to work to earn a livelihood while simultaneously caring for the child with CP. Additionally, the Tunisian population has been severely affected by financial difficulties in recent years, which can increase caregiver burden. Furthermore, in North African countries, most public spaces and transportation systems are not accessible to people with disabilities. These physical obstacles can pose significant challenges for individuals with motor impairments and their caregivers. All these family, economic, and social difficulties may expose mothers of children with CP to high levels of burden.

While fatigue is a pervasive symptom in children with CP, the experience of their primary caregivers, typically mothers, remains largely unexplored. This topic has been infrequently addressed, leaving a significant research gap. Our findings indicate that over half of the participating mothers were severely fatigued, a high prevalence consistent with reports from other populations, such as Turkish mothers of children with CP [19].

Numerous factors may contribute to this maternal fatigue. Interestingly, while Garip *et al*. [19] found no association between fatigue and child-specific parameters (e.g., age, gender, CP type, muscle tone, functional impairment), Pasin *et al*. [40] presented a contrasting view. Their study concluded that a child's functional abilities are crucial determinants of maternal fatigue severity, noting reductions in fatigue with improved gross motor and eating/drinking skills. This divergence may stem from the impact of functional independence; improved self-care capabilities can significantly lessen the physical and emotional burden on mothers. Beyond child-specific characteristics, Davis *et al*. [41] identified additional contributing factors, including lack of social support, marital stress, limited personal freedom, inadequate coping strategies, insufficient physical activity, sleep interruption, employment challenges, and financial strain. Despite these insights, the precise determinants of fatigue in family caregivers remain incompletely understood, highlighting the critical need for further research.

Fatigue also has a strong association with common psychiatric symptoms, particularly depression [42], a consistent finding in relevant research. This well-documented temporal interdependence suggests that fatigue and depression may mutually influence each other or share common underlying risk factors. Furthermore, fatigue and SDs are frequently co-occurring experiences for mothers of children with CP. While Albayrak *et al*. [5] reported a positive correlation between maternal fatigue and sleep quality in this population, our current study observed no significant association between these two parameters. This discrepancy could be attributed to various factors, including our relatively smaller sample size.

The high prevalence and complex nature of caregiver fatigue underscore its critical importance. Evaluating this fatigue is essential because it profoundly impacts the well-being of the family caregiver, which can, in turn, significantly compromise the quality of care provided to the child.

**Limitations:** this study has several limitations that should be acknowledged. First, as a single-center study, the results may not be generalizable to the entire population, which limits the study's strength. Second, the cross-sectional design did not include a control group, making it difficult to determine the direction or causality of relationships. Additionally, the small sample size may have limited statistical power. Furthermore, the majority of participants were mothers of children with spastic CP. To obtain more generalized results, future studies should include mothers of children with ataxia and dyskinetic types of CP. Another limitation is that we only assessed care burden, fatigue, psychological well-being, and sleep quality scores from mothers. However, the presence of motor impairments in children may also influence physical and psychological well-being among fathers. Finally, in this study, caregiver SDs were measured using the PSQI, a subjective questionnaire that may overestimate these parameters. Future studies using objective measures such as actigraphy would be valuable.

## Conclusion

Despite the limitations outlined previously, this study has demonstrated that the caregiving routine for individuals with CP can be physically and emotionally demanding, leading to varying levels of stress, depression, anxiety, and sleep disturbances among mothers. Consequently, healthcare professionals and social workers should be more attentive to the needs of mothers who often silently endure the emotional and physical strains of caregiving. Sleep quality and quantity should be routinely assessed in primary care because they are associated with quality of life, including symptoms of fatigue, energy levels, daytime sleepiness, mental and physical functioning, family relationships, and even bodily pain. In order to reduce caregiver burden and decrease symptoms of depression in parents of children with CP, it is necessary to consider measures that could effectively improve family, social, and financial support.

### What is known about this topic

- Cerebral palsy (CP) is a neurodevelopmental condition that begins in early childhood and persists throughout a person's life;
- Children with CP often require external assistance to perform daily activities; this assistance is frequently provided by mothers who become primary caregivers.

### What this study adds

- Our findings reveal an alarmingly high prevalence of health challenges among caregivers of children with CP, specifically: 74.6% experiencing sleep disturbances, 53.5% severe fatigue, and clinically significant symptoms of depression (63.4%) and anxiety (53.5%);
- This research distinctly shows that poor sleep quality in these caregivers is significantly associated with their child's epilepsy, the caregiver's age, and their own levels of depression and anxiety;
- Notably, our findings indicate that while care burden and fatigue are prevalent among caregivers of children with CP, they were not significantly associated with their sleep quality, highlighting the complex and multi-faceted nature of the challenges faced by this population.

## References

[1] Wimalasundera N, Stevenson VL (2016). Cerebral palsy. Pract Neurol.

[2] Oskoui M, Coutinho F, Dykeman J, Jetté N, Pringsheim T (2013). An update on the prevalence of cerebral palsy: a systematic review and meta-analysis. Dev Med Child Neurol.

[3] McIntyre S, Goldsmith S, Webb A, Ehlinger V, Hollung SJ, McConnell K (2022). Global prevalence of cerebral palsy: A systematic analysis. Dev Med Child Neurol.

[4] Mushta SM, King C, Goldsmith S, Smithers-Sheedy H, Badahdah AM, Rashid H (2022). Epidemiology of Cerebral Palsy among Children and Adolescents in Arabic-Speaking Countries: A Systematic Review and Meta-Analysis. Brain Sci.

[5] Albayrak I, Biber A, Çaliskan A, Levendoglu F (2019). Assessment of pain, care burden, depression level, sleep quality, fatigue and quality of life in the mothers of children with cerebral palsy. J Child Health Care.

[6] Mc Manus V, Corcoran P, Perry IJ (2008). Participation in everyday activities and quality of life in pre-teenage children living with cerebral palsy in South West Ireland. BMC Pediatr.

[7] Reimer MA, Flemons WW (2003). Quality of life in sleep disorders. Sleep Med Rev.

[8] Carpi M, Cianfarani C, Vestri A (2022). Sleep Quality and Its Associations with Physical and Mental Health-Related Quality of Life among University Students: A Cross-Sectional Study. Int J Environ Res Public Health.

[9] Moncer R, Loubiri I, Melki S, Frigui S, Ouannes W, Ben Abdelaziz A (2024). An update on the access to inpatient rehabilitation facilities across Tunisia in 2023. Tunis Med.

[10] Ko J, Woo JH, Her JG (2011). The Reliability and Concurrent Validity of the GMFCS for Children with Cerebral Palsy. J Phys Ther Sci.

[11] Méthodologie de la mesure de la pauvreté en Tunisie.

[12] Seng BK, Luo N, Ng WY, Lim J, Chionh HL, Goh J (2010). Validity and Reliability of the Zarit Burden Interview in Assessing Caregiving Burden. Ann Acad Med Singap.

[13] Dogan Y, Aslaner H (2023). Evaluation of caregiver burdens of caregivers to individuals with chronic heart failure. J Contemp Med.

[14] Worm-Smeitink M, Gielissen M, Bloot L, van Laarhoven HWM, van Engelen BGM, van Riel P (2017). The assessment of fatigue: Psychometric qualities and norms for the Checklist individual strength. J Psychosom Res.

[15] Kazemeini E, Braem MJ, Moorkens G, Balina S, Kastoer C, Op de Beeck S (2019). Scoring of Hypersomnolence and Fatigue in Patients With Obstructive Sleep Apnea Treated With a Titratable Custom-Made Mandibular Advancement Device. J Clin Sleep Med.

[16] Ibrahim N, Yamout D, Bizri M, Taher A (2023). The Arabic Hospital Anxiety and Depression Scale: validation in a sample of Lebanese patients with cancer. Middle East Curr Psychiatry.

[17] Bjelland I, Dahl AA, Haug TT, Neckelmann D (2002). The validity of the Hospital Anxiety and Depression Scale: An updated literature review. J Psychosom Res.

[18] Buysse DJ, Reynolds CF, Monk TH, Berman SR, Kupfer DJ (1989). The Pittsburgh Sleep Quality Index: a new instrument for psychiatric practice and research. Psychiatry Res.

[19] Garip Y, Ozel S, Tuncer OB, Kilinc G, Seckin F, Arasil T (2017). Fatigue in the mothers of children with cerebral palsy. Disabil Rehabil.

[20] Khanna AK, Prabhakaran A, Patel P, Ganjiwale JD, Nimbalkar SM (2015). Social, Psychological and Financial Burden on Caregivers of Children with Chronic Illness: A Cross-sectional Study. Indian J Pediatr.

[21] Bourke-Taylor H, Pallant JF, Law M, Howie L (2013). Relationships between sleep disruptions, health and care responsibilities among mothers of school-aged children with disabilities. J Paediatr Child Health.

[22] Wayte S, McCaughey E, Holley S, Annaz D, Hill CM (2012). Sleep problems in children with cerebral palsy and their relationship with maternal sleep and depression. Acta Paediatr.

[23] Gallagher S, Phillips AC, Carroll D (2010). Parental stress is associated with poor sleep quality in parents caring for children with developmental disabilities. J Pediatr Psychol.

[24] Tsai SY, Lee WT, Lee CC, Jeng SF, Weng WC (2020). Sleep in mothers of children with epilepsy and its relation to their children´s sleep. Res Nurs Health.

[25] Hemmingsson H, Stenhammar AM, Paulsson K (2009). Sleep problems and the need for parental night-time attention in children with physical disabilities. Child Care Health Dev.

[26] Mörelius E, Hemmingsson H (2014). Parents of children with physical disabilities-perceived health in parents related to the child´s sleep problems and need for attention at night. Child Care Health Dev.

[27] Zhang Q, Song D, Liu Y, Chang L, Li C, Li Y (2022). Sleep quality, caregiver burden, and individual resilience among parents of children with epilepsy. Epilepsy Behav.

[28] Chakroun S, Kammoun I, Anane I, Sallemi S, Trabelsi H, Masmoudi K (2021). Quelle relation entre la qualité de sommeil des enfants épileptiques et de celle de leurs mères?. Médecine Sommeil.

[29] Bagherian B, Nematollahi M, Mehdipour-Rabori R (2021). How Parents Cope with the Care of a Child with Epilepsy: Based upon Grounded Theory. Ethiop J Health Sci.

[30] Poza JJ, Pujol M, Ortega-Albás JJ, Romero O (2022). Melatonin in sleep disorders. Neurologia (Engl Ed).

[31] Corsentino EA, Molinari V, Gum AM, Roscoe LA, Mills WL (2008). Family Caregivers´ Future Planning for Younger and Older Adults With Serious Mental Illness (SMI). J Appl Gerontol.

[32] Wullschleger KS, Lund DA, Caserta MS, Wright SD (1997). Anxiety About Aging: A Neglected Dimension of Caregivers´ Experiences. J Gerontol Soc Work.

[33] Han KS, Kim L, Shim I (2012). Stress and sleep disorder. Exp Neurobiol.

[34] Cheshire A, Barlow JH, Powell LA (2010). The psychosocial well-being of parents of children with cerebral palsy: a comparison study. Disabil Rehabil.

[35] Zuurmond M, O'Banion D, Gladstone M, Carsamar S, Kerac M, Baltussen M (2018). Evaluating the impact of a community-based parent training programme for children with cerebral palsy in Ghana. PLoS One.

[36] Unsal-Delialioglu S, Kaya K, Ozel S (2009). Depression in mothers of children with cerebral palsy and related factors in Turkey: a controlled study. Int J Rehabil Res.

[37] Zanon MA, Batista NA (2012). Quality of life and level of anxiety and depression in caregivers of children with cerebral palsy. Rev Paul Pediatr.

[38] Riemann D, Krone LB, Wulff K, Nissen C (2020). Sleep, insomnia, and depression. Neuropsycho pharmacology.

[39] Frutos M, Báez M, Marquez-Gonzalez H, Jiménez-Marquez A, González H (2016). Prevalence of Burden, Family Dysfunction and Depression in Primary Caregiver of Pediatric Patients with Disabilities. J Fam Med Dis Prev.

[40] Pasin T, Karatekin BD, Pasin O (2023). Chronic fatigue syndrome in caregivers of children with cerebral palsy and affecting factors. North Clin Istanb.

[41] Davis E, Shelly A, Waters E, Boyd R, Cook K, Davern M (2010). The impact of caring for a child with cerebral palsy: quality of life for mothers and fathers. Child Care Health Dev.

[42] Leone SS (2010). A disabling combination: fatigue and depression. Br J Psychiatry.

